# Effect of mindfulness-based stress reduction (MBSR) program on depression, emotion regulation, and sleep problems: A randomized controlled trial study on depressed elderly

**DOI:** 10.1186/s12889-024-17759-9

**Published:** 2024-01-23

**Authors:** Nima Javadzade, Sayed Vahid Esmaeili, Victoria Omranifard, Fatemeh Zargar

**Affiliations:** 1 Student Research Committee, Department of Health psychology, School of Medicine, Isfahan University of Medical Sciences, Isfahan, Iran; 2 Student Research Committee, Department of Occupational Health and Safety Engineering, School of Public Health and Safety, Shahid Beheshti University of Medical Sciences, Tehran, Iran; 3 Department of Psychiatry Behavioral Sciences Research Center, School of Medicine, Isfahan University of Medical Sciences, Isfahan, Iran; 4 Behavioral Sciences Research Center, Department of Health Psychology, School of Medicine, Isfahan University of Medical Sciences, Isfahan, Iran

**Keywords:** Mindfulness-based stress reduction, MBSR, Depression, Emotional regulation, Sleep quality, Elderly

## Abstract

**Background:**

Entering old age is associated with various physical and psychological disabilities. Therefore, the aim of this study is to determine the effect of mindfulness-based stress reduction program on emotion regulation and sleep problems in depressed elderly.

**Methods:**

This study was a clinical trial conducted on 60 elderly individuals with depression using purposive sampling. These elderly were referred by geriatricians and were included in the study based on the inclusion criteria. The participants were randomly assigned to two groups: the Mindfulness-Based Stress Reduction (MBSR) group and the control group. Both groups completed the Geriatric Depression Scale (GDS), the Gratz and Roemer Emotion Regulation Questionnaire, and the Pittsburgh Sleep Quality Index before and after the intervention. The MBSR sessions were held for the experimental group in 8 sessions of 90 min each, once a week. Finally, all the data were analyzed using SPSS software version 26 through descriptive and analytical statistics such as mean and standard deviation, t-tests and mixed analysis of covariance (ANCOVA) with repeated measures.

**Results:**

The results showed that the MBSR intervention led to a significant reduction in depression symptoms (*p* < 0.001) and improvement in emotion regulation and sleep quality (*p* < 0.001) among the elderly participants with depression in the intervention group.

**Discussion:**

The results of this study showed that MBSR can be effective in reducing depression levels, improving emotion regulation, and sleep quality among depressed elderly individuals compared to the control group. Caregivers and psychotherapists of nursing homes can use care programs such as MBSR program to improve the physical and mental condition of the elderly.

**Trial registration:**

First Registration: 13/01/2022, Registration Number: IRCT20211118053099N1, Access: https://www.irct.ir/trial/61207.

## Background

Aging is an inevitable phenomenon that gradually begins in all humans, resulting in changes in the body composition and decreased bodily function with advancing age. With the significant increase in the elderly population worldwide and existing medical advancements, it is expected that the number of elderly individuals will increase more rapidly in the coming decades [1]. According to the United Nations Department of Economic and Social Affairs’ World Population Prospects (2017) report, the share of the population aged 65 years and over in the world was 5.08% (8.122 million), and the share of the population aged 60 years and over was 12.3% (9.9 million) in the world [2]. Furthermore, according to the Statistical Center of Iran, the population over 60 years old constitutes about 9.9% of the country’s population, while the population over 65 years old constitutes 4.6% of the country’s population [3]. Estimates also indicate that by 2030, the world’s elderly population will increase from 9 to 16%, and in Iran, it will increase from 5.6 to 17.5% [4].

Depression is the most common mental disorder among people over 60 years old, with a prevalence estimated between 1 and 35% [5]. This prevalence has been reported to be higher among elderly individuals who are hospitalized or reside in care centers due to physical illnesses or cognitive and physical decline [6]. Another problem and discomfort that typically occur during aging and are associated with the natural process of aging are sleep disorders [7]. Since the importance of sleep for the health and well-being of the elderly is well-known, sleep disorders and the consequences of inadequate and inappropriate sleep can significantly affect their quality of life [8, 9]. More than half of elderly individuals suffer from insomnia, and an annual incidence of 5–8% of insomnia has been reported among the elderly [10].

Among the effective mechanisms in the field of geriatric pathology and mental health of the elderly is the ability to regulate emotions [11]. Emotion regulation is conceptualized as a construct that includes awareness and understanding of emotions, acceptance of emotions, and the ability to control reactive behaviors and act in accordance with desired goals to achieve personal objectives and situational demands [12]. Given that the development of emotion regulation strategies continues uninterrupted throughout life and emotional regulation is associated with mental health [13]. Many studies have shown that older adults have higher abilities for emotion regulation than other age groups due to their greater use of adaptive strategies [11, 13].

In recent years, psychological approaches such as mindfulness-based therapies like MBSR have had a significant impact on the treatment of various psychological stresses and improving the quality of life [14]. In mindfulness-based therapy, individuals are taught to accept their experiences as they are, rather than denying or rejecting unpleasant experiences which are considered non-constructive emotional regulation skills, and to be aware of themselves and their reactions to unpleasant experiences [15]. The MBSR approach is an eight-week program that teaches individuals how to use their inherent abilities to respond more effectively to stress, pain, and illness. Various studies using this approach with older adults have shown that it reduces depression [16, 17], increases physical well-being [18], reduces loneliness [19], reduces stress and anxiety [20], and improves sleep problems [21].

Older adults require more psychological help than others to cope with psychological and physical problems. Therefore, given the increasing population trend of vulnerable elderly people, the impact of environmental, social, and cultural factors on the quality of life of the elderly, and the high costs imposed on healthcare systems by these issues, the existence of short-term educational and therapeutic programs that have a significant impact on the psychological well-being of older adults and increase their hope for life is essential.

Developing countries like Iran are not immune to the challenges posed by an aging population [3]. Although life expectancy has increased, there is a lack of adequate medical, social, and cultural infrastructure to effectively address the needs and concerns of the elderly [22]. Therefore, conducting study is crucial for designing such programs. Hence, it is necessary to conduct studies on the effectiveness of MBSR on variables such as emotion regulation and sleep problems in the elderly population, especially elderly individuals with depression, to provide suitable solutions for reducing problems in the elderly. The current study is important as it includes depressed older adults residing in nursing homes. Therefore, according to the authors’ knowledge, this study may be the first to use the MBSR therapeutic approach to examine changes in the psychological conditions of depressed older adults.

## Methods

**Fig. 1:**
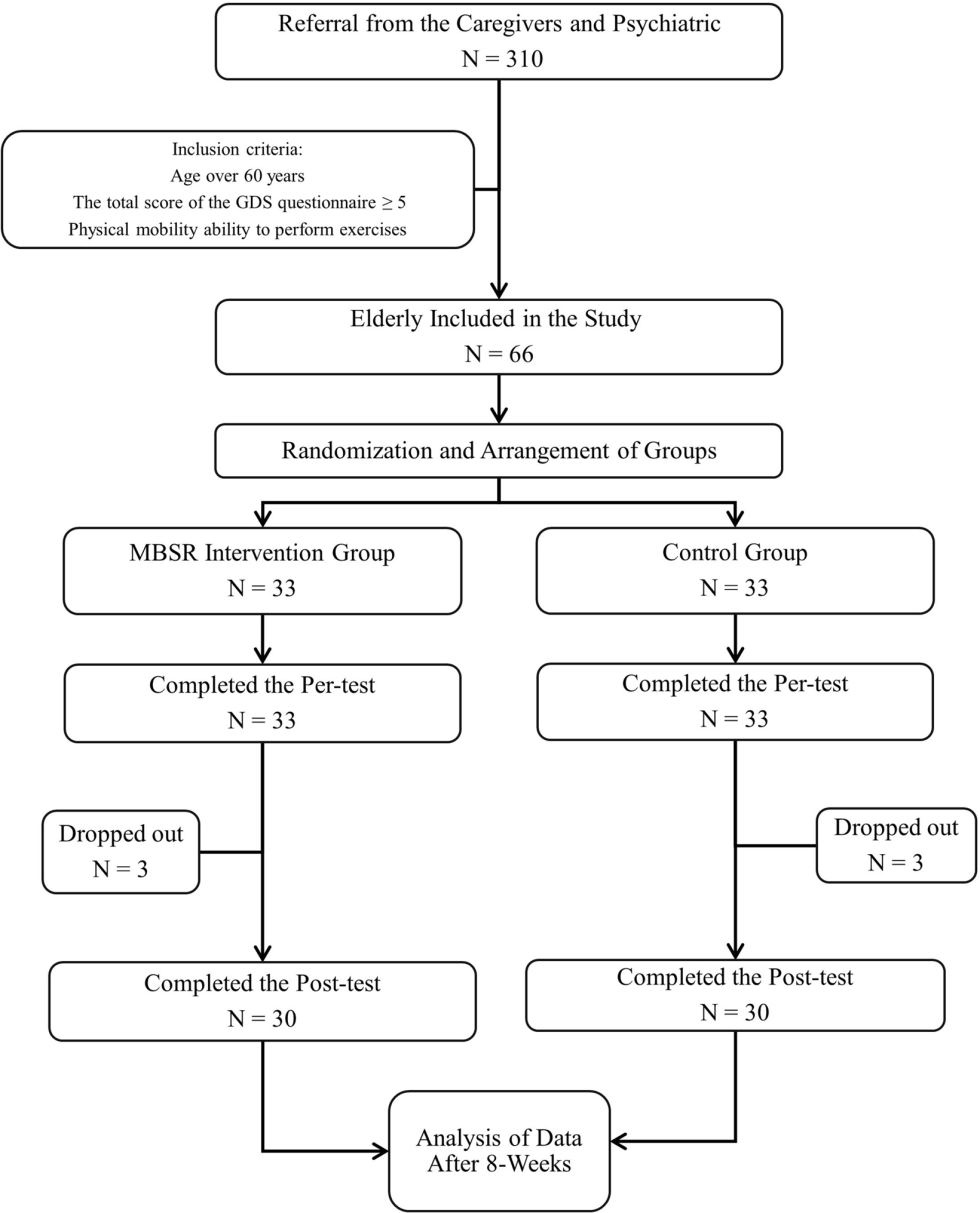
Flow diagram for study participants

This study is a clinical trial conducted on 66 depressed older adults residing in nursing homes in Isfahan. These elderly were referred by geriatricians. In this study, the CONSORT reporting guidelines were used [23]. The sample size of the study was randomly selected based on previous studies [24] and using Eq. (1), considering a 20% dropout rate and dividing the population under study into two intervention and control groups. A summary of the study implementation framework is provided in Fig. 1.

1 $${\text{m}}_{\text{r}\text{e}\text{p}\text{e}\text{a}\text{t}\text{e}\text{d}}=R\bigg\lceil{\left(1+\frac{1}{\lambda }\right)}^{2}\frac{{\left({Z}_{1-\alpha /2}+{Z}_{1-\beta }\right)}^{2}}{{{\Delta }}_{plan}^{2}}+\frac{{Z}_{1-\alpha /2}^{2}}{4}\bigg\rceil$$

### Inclusion and exclusion criteria

The inclusion criteria for this study were: age over 60 years, a total score of 5 or higher on the Geriatric Depression Scale (GDS), ability to participate in at least seven out of eight intervention sessions, no concurrent participation in another study or intervention, absence of other psychiatric disorders, no neurological disorders, physical mobility for performing exercises, willingness to participate in the study, ability to read and write, and the exclusion criteria included withdrawal from participation, absence from more than two sessions, simultaneous development of other chronic illnesses, and use of medications that could interfere with the treatment.

### Measuring tools

After selecting the sample size for the study and assigning them to the intervention and control groups, both groups completed individual information questionnaires including age and gender, as well as depression questionnaires for the elderly (GDS), emotion regulation questionnaire (ERQ), and Pittsburgh Sleep Quality Index (PSQI).

#### The geriatric depression scale

The Geriatric Depression Scale (GDS) questionnaire [17], which is a self-report tool, was used to assess the level of depression symptoms. This test was developed by Yesavage to assess depression in the elderly and is a suitable tool for diagnosing depression symptoms in the elderly, which has been validated in various clinical and non-clinical settings and has good internal and external stability in the clinical diagnosis of depression. The questionnaire consists of 15 questions that are answered in a yes or no format. Scores ranging from 0 to 4 are considered normal, 5–8 indicate mild depression, 9–11 indicate moderate depression, and 12–15 indicate severe depression. This scale has expected psychometric properties. The reliability of this tool has been reported in the elderly population of Iran through alpha, split-half, and retest methods as 0.9, 0.89, and 0.58, respectively [25].

#### Emotion regulation questionnaire

The emotion regulation questionnaire (ERQ) was developed by Gross and John based on a 7-point Likert scale ranging from completely disagree = 1 to completely agree = 7, and examines individual differences in two emotion regulation strategies. Six items assess cognitive reappraisal with questions 1, 3, 5, 7, 8, and 10, and four items assess expressive suppression with questions 2, 4, 6, and 9. Internal consistency of 0.8 and test-retest reliability over a 2-month period were reported for the original version of this questionnaire, and internal consistency of 0.67 and 0.71 were reported for the cognitive reappraisal and expressive suppression subscales, respectively. This tool has been used in different cultures and suitable psychometric properties have been reported for it. The internal consistency reliability of this questionnaire was found to be between 0.48 and 0.68 for reappraisal and between 0.42 and 0.63 for suppression in the University of Milan, Italy [26]. The Persian version of the Gross and John emotion regulation questionnaire has been normalized in Iran by Ghasempour, Ilbeigi, and Hassanzadeh, and its reliability has been reported as alpha 0.6 to 0.81 and validity 0.13 based on internal consistency [13].

#### The Pittsburgh Sleep Quality Index (PSQI)

The Pittsburgh Sleep Quality Index (PSQI) questionnaire was used to assess sleep problems. This questionnaire was developed by Dr. Buysse and colleagues (1989) to investigate sleep problems during the past month, and they obtained an internal consistency of 0.83 using Cronbach’s alpha. The questionnaire consists of seven components, including sleep quality, sleep duration, sleep latency, sleep efficiency, sleep disturbances, use of sleep medication, and daytime dysfunction. It contains 18 self-report questions that are a combination of these seven components, and each component has a range of four Likert-scale levels from 0 to 3. In all cases, “no sleep problem: score of 0, mild sleep problem: score of 1, moderate sleep problem: score of 2, and severe sleep problem: score of 3” are represented [21]. The combination of the scores of the seven components gives a global score, which ranges from 0 to 21, with a score of 0 indicating no sleep problems and a score of 21 indicating multiple problems in all areas. The validity and reliability of this questionnaire have been reported to be 0.86 and 0.89, respectively, and a cut off point of 5 has been considered, with scores higher than this indicating poor sleep quality. In the study by Farahi Moghaddam, the reliability of this questionnaire was reported to be 0.77 using Cronbach’s alpha and its validity was found to be suitable [27].

### MBSR intervention

In this study, MBSR training sessions were held for the experimental group in 8 sessions of 90 min, once a week, and participants were given exercises to practice every week. The control group did not receive any training [28]. The MBSR program was conducted by a certified instructor. One week after the end of the training sessions, both groups completed the study questionnaires as a post-test. The summary of the program and content of the training sessions are presented in Table 1.

**Table 1:** MBSR program outline

Session	Topic/ Theme	Practices
1	Introductions: There is more right with you than wrong with you	Mindful eating, grounding practice, body scan
2	Stress: Our perceptions of our experience impact our mood and physiology	Body scan, mindfulness of the breath
3	Noticing experience and savoring that which is pleasant	Mindful movement, spaciousness practice, mindfulness of sounds
4	Getting unstuck, noticing unhelpful habitual patterns	Mindful movement, short loving kindness, 3-minute breathing space
5	Spaciousness, the lifelong work of moving from reacting to responding	Mindful movement, loving kindness, choiceless awareness, walking meditation
6	Chronic pain deepening the practice on silent retreat	Mindful movement, mindfulness of the breath, loving kindness Mindful movement, body scan, mindfulness of the breath, walking meditation, loving kindness, mountain meditation, mindful eating, walking loving kindness
7	Interpersonal mindfulness, staying open in an unpredictable process	Mindful movement, mindfulness of the breath, walking meditation
8	Forgiveness and moving on, how to support your ongoing practice	Mindful movement, grounding practice, loving kindness

### Statistical analysis

After collecting the data, descriptive statistics of the questionnaires were calculated using descriptive statistical methods such as mean and standard deviation. T-tests analysis were used to compare the ages of participants in both the intervention and control groups. The difference between pre- and post-intervention MBSR scores in both intervention and control groups was examined using t-tests and mixed analysis of covariance (ANCOVA) with repeated measures. Also, prior to conducting the statistical analysis, the normality of the data was examined through the utilization of the Shapiro-Wilk test, with a significance level set at 0.05. All analyses were performed using SPSS 26.0 software (IBM Corporation, Armonk, NY).

## Results

After the intervention, three elderly depressed individuals in the control group withdrew from the study. In the intervention group, two elderly individuals withdrew due to medication intervention and one withdrew due to personal reasons. Finally, the results of data from 60 elderly depressed individuals in the two groups (30 in the intervention group and 30 in the control group) were analyzed. There was no statistically significant difference in age between the MBSR and control groups (*P* > 0.05). However, 60% of each study group consisted of women, and a significant difference was found between the two groups (Table 2).

**Table 2:** Baseline characteristics

Demographics	MBSR Group (*n* = 30)	Control Group (*n* = 30)	P
**Age** (Mean ± SD)	66 ± 5	67 ± 5	0.388
**Sex** (Male/Female)	12/18	12/18	< 0.001

The normality of the data was assessed using the Shapiro-Wilk test. The results indicated that all of the data in both the control and intervention groups exhibited a normal distribution (*P* > 0.05) (Table 3).

**Table 3:** The result of the measurement of data normality using the Shapiro-Wilk test

Variables	Group	Shapiro-Wilk test		
		Statistics	df	P
Depression	Intervention	0.934	29	0.064
	Control	0.951	29	0.178
Emotion regulation- cognitive reappraisal	Intervention	0.934	29	0.064
	Control	0.952	29	0.192
Emotion regulation-expressive suppression	Intervention	0.946	29	0.128
	Control	0.949	29	0.161
Sleep problems	Intervention	0.958	29	0.279
	Control	0.962	29	0.352

According to Table 4, the results of the ANCOVA model showed that the mean difference values created between the control and intervention groups after the intervention were statistically significant at the sample level of 30 after adjusting for the effect of depression values in these groups before the intervention. In addition, the results of this model showed that the mean difference values created between the control and intervention groups after the intervention were statistically significant at the sample level of 30 after adjusting for the effect of emotion regulation values in the two dimensions of reappraisal and suppression in these groups before the intervention. Furthermore, the results of this model showed that the mean difference values created between the control and intervention groups after the intervention were statistically significant at the sample level of 30 after adjusting for the effect of sleep problems values in these groups before the intervention.

**Table 4:** The output of the ANCOVA model for comparing the effects of the study variables

Variable	Time	Average^a^	Standard error	Confidence limits 95%		F	Partial Eta Squared	Observed Power	P^**^
				down	up				
Depression	Intervention	6.284	0.202	5.880	6.689	74.143	0.572	1.000	< 0.001
	Control	8.816		8.411	9.220				
Emotion regulation- cognitive reappraisal	Intervention	22.671	0.349	21.973	23.369	21.796	0.227	0.996	< 0.001
	Control	20.362		19.664	21.061				
Emotion regulation-expressive suppression	Intervention	12.316	0.274	11.768	12.865	73.443	0.563	1.000	< 0.001
	Control	15.650		15.102	16.199				
Sleep problems	Intervention	11.580	0.191	11.198	11.961	43.314	0.432	1.000	< 0.001
	Control	13.354		12.972	13.735				

^**^Significant *P* Values

^a^Estimated Marginal Means

## Discussion

In recent years, therapeutic methods such as MBSR have gained attention not only as a method to reduce stress, but also to improve chronic physiological symptoms [28, 29]. Numerous studies have been conducted on the physiological mechanisms of MBSR and its potential effectiveness in reducing depression, regulating emotions, alleviating anxiety, and chronic pain [19, 30]. On the other hand, study on MBSR shows that it has the potential to improve the health of older adults and the elderly, especially in terms of depression, sleep problems, and overall mental health [19, 31]. Therefore, the present study aimed to determine the effectiveness of MBSR on variables such as depression, emotion regulation, and sleep problems in the depressed elderly community.

The results of the present study on the effect of MBSR on improving depression in the elderly, 60% of whom were women, showed that MBSR has a significant effect on improving depression in the elderly. After 8 sessions of training for depressed elderly individuals in the intervention group, there was a reduction in the level of depression symptoms compared to the elderly in the control group. The study by Kumar et al. also showed a significant reduction in depression among the elderly after MBSR training [30]. Therefore, systematic reviews and meta-analyses have examined the role of MBSR in a wide range of patient and psychological problem groups, including depression [1, 32, 33]. However, the level of depression in the study group before entering the intervention and control groups in previous MBSR studies has not been considered. Therefore, it can be concluded that in mindfulness programs such as MBSR, individuals are encouraged to take a non-judgmental approach to their mental and emotional content, which can reduce their depression.

The present study also demonstrated that MBSR has a significant effect on reducing emotional regulation in the dimensions of reappraisal and suppression. The findings are consistent with the results of the studies by Marciniak et al. [34]. and Serpa et al. In the Marciniak et al. study, the mean age of the participants was higher than in the present study, but like the present study, the majority of participants were women. The results of the study by Hatamian et al. on elderly individuals with heart disease also showed that psychological treatment approaches such as MBSR can be effective in regulating emotions and reducing anxiety sensitivities [13]. The interpretation of the results of the MBSR intervention in this study can be based on Gross’s emotion regulation model. Similar processes of reappraisal can influence emotional responses. Attempts to reduce emotion through reappraisal alter the entire course of the emotional response and lead to fewer experiential, behavioral, and physiological responses. In contrast, mindfulness includes observing, describing, and allowing emotions without judgment or trying to control them. This approach can impact habitual or automatic responses to emotional behaviors and the evaluations associated with them. Therefore, the findings suggest that MBSR is an effective and preventive strategy for reducing high levels of anxiety and increasing emotion regulation strategies, which can help depressed elderly individuals reduce their anxiety using mindful approaches during emotional experiences and regulate and manage them effectively [35].

On the other hand, the results of the present study showed that MBSR is an effective therapeutic approach for sleep problems in the elderly, as measured by the PSQI scale. The findings of the present study are similar to some of the positive results obtained from several mindfulness-based therapy studies for individuals with sleep problems and insomnia. A meta-analysis of 16 studies showed that mindfulness-based interventions can lead to improved sleep in various groups, and this improvement can continue for 2–6 months after treatment initiation [36]. The results of studies by Jones et al. [37]. and Wang et al. on university rowers and breast cancer patients, respectively, also support these findings. However, the results of a large meta-analysis contradict these findings and suggest that MBSR may be ineffective in improving sleep quality in patients with chronic insomnia and cancer, which may be due to the small sample size in the studies under investigation [38].

Furthermore, from a cognitive-behavioral perspective, sleep problems are caused by automatic arousal, dysfunctional cognitions, and distress resulting from these factors. The MBSR intervention addresses these issues by increasing attentional control over the autonomic nervous system, leading to reduced worry, rumination, and mood disorders. In conclusion, the MBSR intervention is an effective therapeutic approach for reducing limited awareness and disturbing behaviors while promoting acceptance of experiences and living in the moment. This approach can positively impact arousal and reactivity processes, leading to improved sleep quality, daily functioning, and sleep-wake perception in the elderly.

This study, like other studies, also had some limitations. The main limitation is that the assessments were mostly self-reported by depressed elderly individuals based on a self-report scale. Given that elderly individuals with psychological problems often estimate their actual condition incorrectly, this may introduce bias into the study results. However, more objective measures such as polysomnography (PSG) can provide more accurate results for sleep problems. Nevertheless, attention should be paid to the costs and additional burden on participating elderly individuals. Since the target population included depressed elderly individuals, the results cannot be generalized to the general elderly population. Additionally, the lack of follow-up and small sample size are other limitations of this study. However, the sample size was sufficient to achieve a statistical effect of MBSR. It is suggested that this study be conducted among other populations to better generalize the results. Furthermore, it is recommended that the follow-up stage be given more attention in future studies, and health professionals such as nurses, rehabilitation specialists, psychologists, and caregivers who work with elderly individuals with psychological problems should pay special attention to the role of psychological variables and use this side-effect-free therapeutic approach as a very effective complement to drug therapies to reduce the problems of the elderly.

## Conclusion

Overall, the results of this study indicate the effectiveness of mindfulness-based stress reduction in improving cognitive health, such as depression, emotion regulation, and sleep problems, in depressed elderly individuals residing in nursing homes after a period of 8 weeks. also, The study findings indicate that the MBSR intervention is effective in the elderly, despite there being no significant age or gender differences between the control and intervention groups. Therefore, this intervention can be used as a complementary method of medical treatment to improve the psychological health of these patients. Overall, mindfulness-based interventions familiarize the individual with psychological problems and coping mechanisms, and by neutralizing the effects of some problems and disorders, they can help improve physiological and psychological functioning. Therefore, it seems that by using mindfulness-based stress reduction training to provide services to elderly individuals with a history of or current depression, the severity of their disorders and illnesses can be reduced. Therefore, the MBSR approach is recommended as an effective method for reducing various psychological problems and improving the well-being of the elderly, and it should be considered by managers and policymakers in the field of psychology and psychotherapy for use in the elderly community.

## Data Availability

The datasets generated and/or analyzed during the study are not publicly available and the authors can provide the data upon reasonable request.
